# Nanomaterials as a Tool for Increasing Sensitivity and Selectivity in the Analytical Chemistry of Tungsten by Stripping Voltammetry

**DOI:** 10.3390/ma19061202

**Published:** 2026-03-18

**Authors:** Malgorzata Grabarczyk, Edyta Wlazlowska

**Affiliations:** Department of Analytical Chemistry, Institution of Chemical Sciences, Maria Curie−Sklodowska University, 20-031 Lublin, Poland; malgorzata.grabarczyk@mail.umcs.pl

**Keywords:** tungsten, cathodic stripping voltammetry, electrochemical sensor, real samples, certified reference materials, carbon nanotubes

## Abstract

Tungsten is an extremely durable metal with a wide range of industrial applications and its toxicity is relatively low, although chronic exposure to its compounds can lead to adverse health effects. This paper proposes a method for the determination of trace amounts of tungsten using cathodic stripping voltammetry (CSV). A hybrid structure based on a mixture of multi-walled carbon nanotubes and spherical glassy carbon was used as the working electrode, on the surface of which a film of lead was formed during the measurement to increase the efficiency of the determination. A comprehensive optimization of the analytical parameters, including accumulation potential and time, signal recording conditions and electrolyte solution composition, was carried out to maximize sensitivity and improve the signal-to-noise ratio. The method developed achieved a detection limit for tungsten of 3 × 10^−10^ mol L^−1^, demonstrating its high sensitivity. The working electrode showed selectivity, signal reproducibility and resistance to the presence of potential interferences. The reliability and applicability of the proposed solution were confirmed by applying the method to the analysis of real environmental samples and certified reference materials, with satisfactory results. The presented analytical procedure represents a promising tool for the routine determination of tungsten in complex real matrices.

## 1. Introduction

Electrochemical methods are one of the key groups of analytical techniques used in modern analytical chemistry. Their rapid development is driven by continuous technological advances in instrumentation and growing analytical needs in various fields of science and industry. Among electrochemical methods, stripping voltammetry is considered particularly important because of its exceptionally high sensitivity and ability to determine trace concentrations of analytes in real samples. Because of these properties, this technique is widely used in environmental, biological, pharmaceutical and industrial analysis [1,2].

The ongoing miniaturization of analytical instrumentation, including the development of portable electrodes and compact measurement systems, enables in situ analysis, significantly increasing the functionality and flexibility of electrochemical methods in field conditions [3,4]. An additional advantage is the short duration of the analysis and the simplicity of the measurement procedures, which contribute to the growing popularity of these techniques [3,4,5]. Stripping voltammetry includes several variants, such as anodic (ASV), cathodic (CSV) and adsorptive stripping voltammetry (AdSV), which differ mainly in the mechanism of analyte preconcentration prior to the measurement step. The choice of a particular variant depends on the properties of the analyte, the composition of the sample matrix and the required sensitivity of the determination [6,7,8].

The type of electrode used in stripping voltammetry is crucial, as it affects the sensitivity and accuracy of the measurements, as well as the range of analytes that can be determined. Its physicochemical properties determine the efficiency of analyte concentration, the quality of the analytical signal and the resistance to matrix interference [9]. In recent years, so-called film electrodes have gained particular attention as a modern alternative to classic mercury electrodes [10]. They combine high analytical efficiency with a favourable environmental and toxicological profile, making them an attractive choice in modern electroanalysis [10,11]. Film electrodes usually consist of a conductive substrate on which a thin layer (film) of metal or other electrochemically active material is deposited [10]. In the design of film electrodes, the choice of substrate material on which the active layer is deposited is of great importance. Glassy carbon, graphite and carbon paste electrodes are the most commonly used for the application of metal films in stripping voltammetry [12]. These materials have good conductivity, a wide potential window and chemical stability [10,11,12]. Carbon nanomaterials, particularly carbon nanotubes (CNTs), have attracted considerable attention in modern electrode design [11]. Their high specific surface area, excellent electrical conductivity, and strong adsorption capability enhance analyte accumulation and facilitate charge-transfer processes. Recent studies have demonstrated that nanostructured and roughened electrode surfaces significantly improve sensitivity in stripping voltammetry by increasing the effective electroactive area and accelerating electron transfer [13,14]. The integration of CNTs with conventional substrates therefore leads to lower detection limits, improved linearity, and enhanced resistance to matrix effects. The combination of multi-walled carbon nanotubes with spherical glassy carbon provides a conductive and mechanically stable composite with a large electroactive surface area, which enhances analyte accumulation and improves the sensitivity of electrochemical detection [15].

Depending on the specific nature of the analyte and the measurement conditions, various metals or their compounds with appropriate electrochemical properties are used to form film layers in working electrodes [16]. The most commonly used film materials are metals such as bismuth (Bi), antimony (Sb), gold (Au), lead (Pb) and, in classic applications, mercury (Hg) [17]. The key criteria for selecting the film material are its ability to form stable amalgamates or complexes with analytes, a wide working potential window, good solubility of analytes in the metal layer and low electrochemical background noise [12,16,17,18,19].

Although mercury has historically been the standard in electroanalysis due to its unique physico-chemical properties, it is now being phased out of analytical applications due to toxicity and environmental restrictions [18]. Bismuth is increasingly being used instead, offering similar electrochemical performance with a much more favourable ecotoxicological profile [20]. Antimony is valued for its good compatibility with ASV and AdSV techniques and its resistance to interference from the sample matrix [21]. Gold is mainly used in the analysis of compounds containing thiol groups and other organic substances capable of forming stable complexes with its surface [22]. Lead is increasingly investigated as a film material due to its favorable electrochemical properties and suitability for trace metal analysis [23].

Lead film electrodes (PbFE) are a promising alternative to traditional mercury electrodes in stripping voltammetry techniques, offering high sensitivity and selectivity while eliminating toxic mercury [19]. They are usually made by depositing a thin layer of lead on a conductive surface, usually glassy carbon. Depending on the experimental approach, the lead film can be formed in situ by electrodeposition of Pb(II) ions from the analytical solution prior to the measurement step, or ex situ where the film is prepared in a separate solution before the analytical procedure. The in situ method is widely used because it simplifies the experimental procedure; however, the ex situ approach may allow better control over the thickness and homogeneity of the deposited film [24,25]. The choice between in situ and ex situ methods depends on the specific nature of the analysis, the equipment available and the requirements for sensitivity and repeatability of the measurements. Both techniques have their advantages and limitations and their application should be tailored to the specific analytical case [19,24,25].

Tungsten (W) is a chemical element in the transition group of metals, best known for its exceptional hardness and the highest melting point of all metals (over 3400 °C) [26,27]. It also has high density, corrosion resistance and very good thermal and electrical conductivity. Owing to these properties, tungsten is widely used in a variety of industrial applications, including electronics, aerospace technology, and the production of specialized alloys [26,27]. In chemical compounds, it exists in various oxidation states from −II to +VI but its most stable and common forms are those with an oxidation state of +VI [28]. In the natural environment, tungsten occurs mainly in the form of minerals—scheelite (CaWO_4_) and wolframite ((Fe,Mn)WO_4_). Its natural concentration in soil and water is usually low, but the intensification of its industrial use is leading to an increase in its presence in the environment, making it a new and significant environmental pollutant [29,30].

Consequently, the occurrence of tungsten in environmental matrices has attracted increasing scientific attention. Despite the growing interest in this element, our knowledge of its full toxicological profile remains limited. In addition, there is increasing evidence that tungsten may act synergistically with other heavy metals, such as cobalt, to enhance their toxicity [27,29]. Such interactions can lead to more serious health effects, including respiratory, cardiovascular, skeletal and immune system disorders [31,32]. In this context, monitoring the presence of tungsten in the environment and in living organisms is of particular importance [27,29,30,31,32]. Its concentrations can be determined using modern analytical techniques such as mass spectrometry, spectroscopy and voltammetry, which can detect even trace amounts of this element.

The scientific literature is dominated by studies on the determination of tungsten using advanced instrumental techniques such as spectrofluorimetric [33,34,35,36], spectrophotometry [37,38], atomic absorption spectrometry (AAS) [39,40], inductively coupled plasma mass spectrometry (ICP-MS) [41,42,43] and inductively coupled plasma atomic emission spectroscopy (ICP-AES) [44,45]. Although these methods provide high sensitivity and accuracy, they often require expensive instrumentation and complex sample preparation procedures, frequently involving decomposition in strong acids. These factors limit their routine application, particularly in less well-equipped laboratories. As a result, there is a growing interest in alternative methods for tungsten determination that offer lower cost, simpler sample preparation and high analytical sensitivity [41,42,43]. An example of such a technique is stripping voltammetry, which enables the determination of trace amounts of tungsten in various environmental and biological matrices. Unfortunately, despite its great potential, the number of scientific publications using stripping voltammetry for the determination of tungsten is still limited. The most recent work on tungsten determination has been developed using cathodic stripping voltammetry (CSV) [46], whereas earlier work was mainly based on adsorption stripping voltammetry (AdSV) [47,48,49,50,51,52]. The diversity of stripping voltammetry techniques allows the method to be adapted to different analytes and sample matrices. We therefore attempted to develop a new method for the determination of tungsten using a working electrode that had not previously been used for this purpose.

The most commonly used working electrodes for tungsten determination have been the hanging mercury drop electrode (HMDE) [47,48,49,50], the silver-based mercury film electrode (Hg(Ag)FE) [51], the modified glassy carbon electrode (GCE) [52] and the fixed bismuth microelectrode [46]. Despite their good analytical performance, these electrodes have certain limitations, such as the use of mercury-based materials and relatively complex preparation procedures. Therefore, developing alternative electrode materials with improved analytical properties remains of considerable interest. In this study, a composite electrode consisting of a mixture of multi-walled carbon nanotubes (MWCNTs) and spherical glassy carbon powder (SGC). Literature reports indicating improved analytical performance as a result of modification of the glassy carbon electrode with lead film provided the basis for carrying out an analogous modification of the electrode used in the method developed [52]. The aim of this study was to evaluate whether this electrode configuration could enhance the sensitivity and selectivity of tungsten determination.

This study presents the use of a novel MWCNTs/SGC composite electrode modified in situ with a lead film for the determination of tungsten(VI) by cathodic stripping voltammetry. This modification strategy is based on previous reports demonstrating the improved analytical performance of lead film-coated carbon electrodes [52]. The experimental conditions were systematically optimized, including the type and concentration of the supporting electrolyte, the concentration of Pb(II), the accumulation potential and time, and the square-wave parameters. The analytical performance of the method was evaluated in terms of its calibration characteristics, repeatability and selectivity in the presence of potential inorganic and organic interferents. The applicability of the proposed procedure was verified by analyzing real water samples.

## 2. Experimental

### 2.1. Apparatus

A μAutolab analyzer (Eco Chemie, Utrecht, The Netherlands) was employed for electrochemical techniques. A conventional three-electrode system with an Ag/AgCl reference electrode, a platinum counter electrode, and multiwall carbon nanotube/spherical glassy carbon (MWCNT/SGC) electrode as a working electrode. All measurements were carried out using a quartz cell. The surface of the working electrode array was polished once a day directly before the voltammetric measurements using an abrasive paper of 2500 grit and then with paste from 0.3 µm Al_2_O_3_ on a Buehler polishing pad. After polishing, the electrode was cleaned with deionized water in an ultrasonic bath for about 30 s. All electrodes were fabricated and assembled at the Department of Analytical Chemistry, Maria Curie-Sklodowska University (Lublin, Poland). An Orion Star A211 pH benchtop meter (Thermo Scientific, Waltham, MA, USA) was used to measure the pH values of the solutions.

### 2.2. Chemicals

All used chemical reagents were of analytical reagent grade or Suprapur. Deionized water obtained from Milli-Q system purification was used for the preparation of all solutions. Acetate buffer of 1 mol L^−1^ and pH 5.3 was prepared from acetic acid (CH_3_COOH) and sodium hydroxide (NaOH) using Suprapur reagents purchased from Merck (Darmstadt, Germany). For this purpose, 57.1 mL of 17.5 mol L^−1^ CH_3_COOH solution was added to approximately 800 mL of deionized water. Then, 10 mol L^−1^ NaOH solution was added to the mixture to obtain a final pH of 5.3. Finally, the volume of the solution was made up to 1 L with water. A standard solution of 1 g L^−1^ of W(VI) were procured from Fluka (Buchs, Switzerland). Working solutions of W(VI) with concentrations of 1 × 10^−4^, 1 × 10^−5^ and 1 × 10^−6^ mol L^−1^ were prepared by appropriate dilution of the standard solution in 0.01 mol L^−1^ HNO_3_, adjusting the concentration to the analysis requirements. Standard solutions of Pb(II) at a concentration of 1.0 g L^−1^ and other inorganic ions used in an interference study were purchased from Sigma-Aldrich (St. Louis, MO, USA). A standard solution of 1.0 g L^−1^ Triton X-100 and cetyltrimethylammonium bromide (CTAB) were acquired from Fluka (Buchs, Switzerland). Humic acid sodium salt (HA) was purchased from Sigma-Aldrich (St. Louis, MO, USA). Natural organic matter (NOM) and river fulvic acid (FA), originating from the Suwannee River, were purchased from the International Humic Substances Society. For evaluation of the accuracy of measurements, the certified reference materials SPS-SW1 Reference Material for Measurement of Elements in Surface Water and SPS-WW1 Reference Material for Measurement of Elements in Wastewaters from Spectrapure Standards, Oslo, Norway, were used. Multi-walled carbon nanotubes (MWCNTs; O.D. 10 ± 1 nm, I.D. 4.5 ± 0.5 nm, length 3–6 µm) were supplied by Sigma-Aldrich (St. Louis, MO, USA). Spherical glassy carbon powder (0.4–12 µm) was purchased from HTW Hochtemperatur-Werkstoffe GmbH (Thierhaupten, Germany). Araldite F epoxy resin (CY205) with ARADUR HY 905 hardener (Huntsman, Basel, Switzerland).

### 2.3. Construction of the MWCNT/SGC Electrode

The working electrode was fabricated by initially mixing multi-walled carbon nanotubes (MWCNTs) with an epoxy resin in a weight ratio of 1:25, resulting in a homogeneous composite. This composite was then subjected to a temperature of 115 °C and hot centrifugation to eliminate any entrapped air bubbles. Afterward, the de-aerated material was blended with spherical glassy carbon (SGC) powder, with particle sizes ranging from 0.4 to 12 µm, in a 2:1 mass ratio. The resulting mixture was introduced into a polyether ether ketone (PEEK) tube with a 2 mm diameter opening and compacted under pressure. A copper wire was incorporated to ensure proper electrical conductivity. Following compaction, the surface of the newly formed electrode was initially polished with 120-grit sandpaper, followed by finer polishing using 2000-grit sandpaper. To remove residual abrasive material, the electrode was thoroughly rinsed with deionized water and sonicated for 30 s in an ultrasonic bath (Sonic-3, Polsonic, Warsaw, Poland). The procedure of preparing the MWCNT/SGC electrode was described in detail in [15], which also included a microscopic image and a picture showing the construction of the electrode.

### 2.4. Measurement Procedures

**Figure 1:**
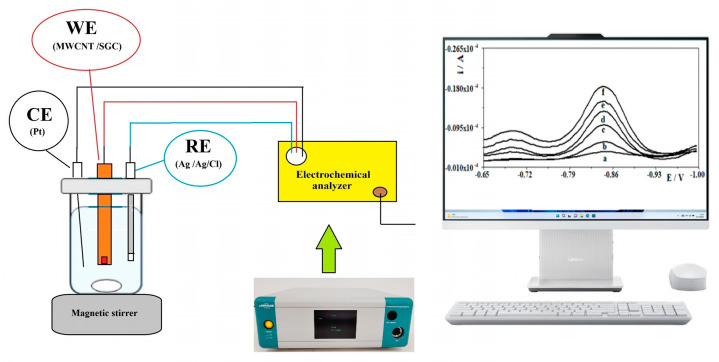
Schematic diagram of the electrochemical measurement setup used in this study. The labels (a–f) correspond to the following frequency values: (a) 100 Hz, (b) 150 Hz, (c) 200 Hz, (d) 250 Hz, (e) 300 Hz and (f) 400 Hz.

Electrochemical measurements were performed using the three-electrode system described above. A schematic representation of the experimental setup is shown in Figure 1. The sample to be analyzed was placed in a measuring cell and then 1 mL of acetate buffer with a concentration of 1.0 mol L^−1^ (pH 5.3) and an appropriate amount of Pb(II) solution was added to give a final concentration of Pb^2+^ ions of 7.24 × 10^−5^ mol L^−1^. Then, the volume of the solution was made up to 10 mL with distilled water. Solutions were not deoxidized prior to measurement and all measurements were performed at room temperature. The surface of the working electrode (MWCNTs/SGC) was cleaned electrochemically by applying a potential of −1.3 V for 15 s and a potential of +0.2 V for 10 s, relative to the Ag/AgCl reference electrode. At a potential of −1.3 V, the residues from the previous measurement were reduced to a metallic state and then broken off the electrode at a potential of +0.2 V. Then, at a potential of −0.7 V, the simultaneous formation of a lead film and the accumulation of tungsten ions on the surface of the working electrode was carried out. The accumulation time was 80 s. A stabilization period of 5 s at a potential of 0.2 V (standby potential) was applied at the end of the accumulation phase. The analytical signal was then recorded using square wave voltammetry (SWV) in the potential range −0.65 V to −1.0 V. The square wave parameters were as follows: frequency 400 Hz, pulse amplitude 40 mV and step potential 8 mV. All measurements were performed in triplicate under identical conditions, and the reported results represent the mean (*n* = 3).

According to previously reported polarographic data and the characteristic half-wave potentials of tungsten as described in the literature, the detection mechanism of the proposed SWV-based stripping procedure can be explained as follows. During the accumulation step at −0.7 V, tungsten are preconcentrated electrochemically at the surface of the freshly formed lead film, most likely in the W(V) oxidation state. During the subsequent SWV scan (−0.65 to −1.0 V), the accumulated W(V) species undergo further reduction to W(III) [53,54]. This redox transformation produces a well-defined cathodic peak, which forms the basis for quantitatively determining W(VI).

## 3. Results and Discussion

### 3.1. Impact of pH and Solution Concentration on the Electrochemical Response

The correct choice of supporting electrolyte is key to optimizing the method of determining trace amounts of tungsten(VI) using cathodic stripping voltammetry (CSV). The appropriate electrolyte should provide stable redox conditions, allow effective accumulation of the analyte on the electrode surface and minimize measurement background interference, which directly affects the sensitivity and selectivity of the method. In the literature, tungsten has most often been determined in the presence of complexing agents, which requires the use of an acidic environment, usually with a pH in the range of 2.0–3.3 [46,50,51,52]. Under these conditions, tungsten(VI) complexes were formed which could be adsorbed on the surface of the working electrode, increasing the efficiency of the accumulation process.

**Figure 2:**
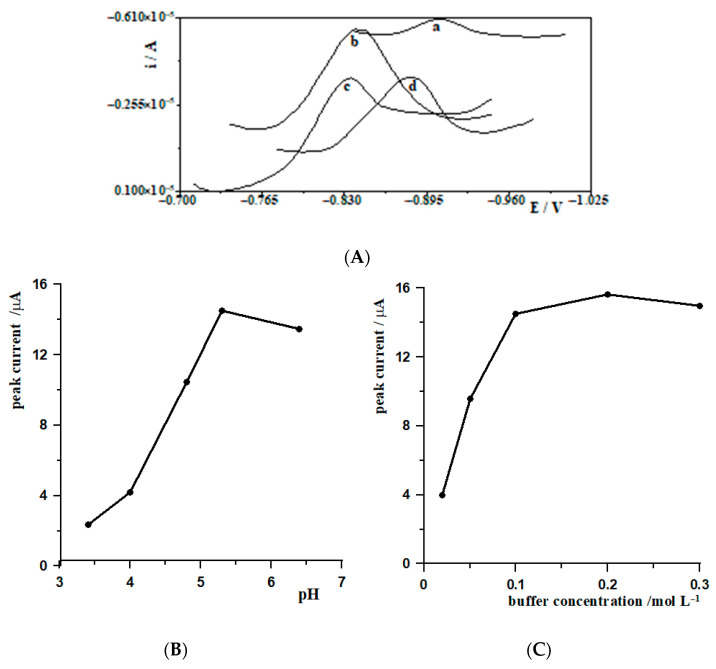
(**A**) Comparison of the CSV curves registered for 5 × 10^−7^ mol L^−1^ W(VI) at a MWCNTs/SGCE in different supporting electrolytes: (a) 0.1 mol L^−1^ Britton–Robinson buffer (pH 4.5), (b) 0.1 mol L^−1^ acetate buffer solution (pH 5.3), (c) 0.1 mol L^−1^ hydrochloric acid, and (d) 0.1 mol L^−1^ acetic acid. (**B**) The effect of supporting electrolyte pH, and (**C**) acetate buffer concentration on the peak current response of W(VI). Accumulation conditions: −0.7 V for 80 s. Measurement parameters: frequency 400 Hz, amplitude 40 mV, and step potential 8 mV.

In this study, a MWCNTs/SGCE working electrode and an analytical solution containing tungsten(VI) at a concentration of 5 × 10^−8^ mol L^−1^ and lead ions at a concentration of 7.23 × 10^−5^ mol L^−1^ were used. Four different supporting electrolytes were tested to optimize the analytical conditions: Britton-Robinson buffer, acetate buffer, acetic acid and hydrochloric acid. Each electrolyte was added in a volume of 1 mL to a measuring vessel containing 10 mL of analytical solution. Peak current analysis showed significant differences between the electrolytes tested. The highest signal was recorded for the acetate buffer, indicating the highest efficiency in the accumulation and reduction of tungsten(VI). The influence of the type of electrolyte on the potential for the appearance of an analytical signal was also observed. In the Britton-Robinson buffer, the peak appeared at a more negative potential, which may be due to the higher pH and lower proton activity. In the case of more acidic solutions (HCl and acetic acid), the signal was shifted towards more positive potentials, corresponding to the characteristics of an environment with a higher concentration of H^+^ ions. Figure 2A illustrates the signals obtained for different supporting electrolytes. Based on the results obtained, it was concluded that acetate buffer is the most suitable supporting electrolyte for the determination of tungsten(VI) in the electrochemical system tested.

In the next part of the study the effect of the pH of the acetate buffer (range 3.4–6.4) on the analytical signal level was analyzed. Increasing the pH from 3.4 to 5.3 resulted in an increase in the peak current, reaching a maximum at pH 5.3. A further increase in pH to 6.4 resulted in a decrease in the signal, probably due to reduced adsorption efficiency of tungsten(VI) or its partial hydrolysis. The dependence of the W(VI) peak height on the pH of the acetic acid buffer is shown in Figure 2B.

The next parameter analyzed was the concentration of acetate buffer at a constant pH of 5.3. The range from 0.02 mol L^−1^ to 0.3 mol L^−1^ was tested. Increasing the concentration resulted in an increase in the peak current. However, a significant broadening of the peaks was observed at concentrations of 0.2 mol L^−1^ and 0.3 mol L^−1^, which could have made accurate determination of the potential difficult and reduced signal resolution. The most favourable concentration of acetate buffer for the determination of tungsten by the CSV method, giving a high, narrow and well-formed signal peak, was 0.1 mol L^−1^ (Figure 2C). In conclusion, an acetate buffer with a pH of 5.3 and a concentration of 0.1 mol L^−1^ was found to be the optimal supporting electrolyte for the determination of tungsten(VI) by CSV on a MWCNTs/SGC electrode modified with a lead film. It provides a compromise between high determination sensitivity and good resolution and analytical signal quality.

### 3.2. Impact of Pb(II) Concentration

In this study, an attempt was made to use a MWCNTs/SGC electrode modified in situ by the formation of a lead film to evaluate its suitability for the determination of tungsten(VI). In this study, a MWCNTs/SGC electrode modified in situ by lead film formation was evaluated for the determination of tungsten(VI). Preliminary experiments showed that no analytical signal was obtained when only the supporting electrolyte and analyte were present. The addition of Pb(II), enabling in situ formation of a lead film on the electrode surface, was essential for effective analyte accumulation and signal generation. Therefore, optimization of the Pb(II) concentration was necessary to ensure the formation of a stable and active film suitable for tungsten(VI) determination.

**Figure 3:**
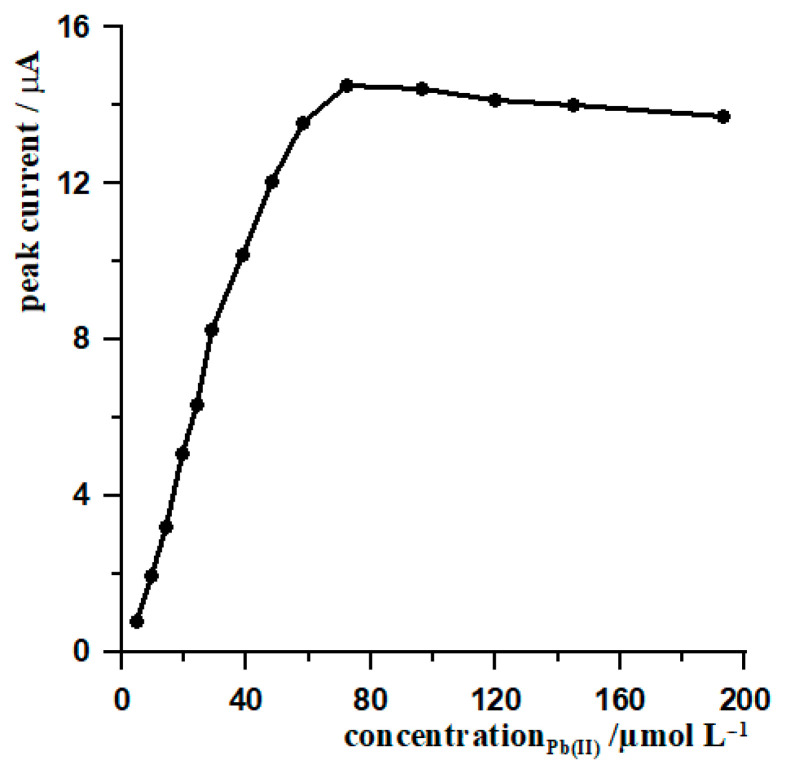
The effect of Pb(II) concentration on the peak current response of 5 × 10^−7^ mol L^−1^ W(VI). Accumulation conditions: −0.7 V for 80 s. Measurement parameters: frequency 400 Hz, amplitude 40 mV, and step potential 8 mV.

In order to determine the optimum concentration of Pb(II), a series of measurements were carried out with different concentrations of lead ions ranging from 4.83 × 10^−6^ mol L^−1^ to 1.93 × 10^−4^ mol L^−1^. The solution also contained tungsten ions at a concentration of 5 × 10^−8^ mol L^−1^ and an acetate buffer at a concentration of 0.1 mol L^−1^ and pH 5.3. The results obtained showed a clear increase in signal height with increasing Pb(II) concentration. The peak current increased to a concentration of 7.24 × 10^−5^ mol L^−1^ and reached its maximum value. Further increases in the lead ion concentration did not result in an increase in the signal; on the contrary, a gradual decrease was observed. Figure 3 shows the relationship between the change in the tungsten signal and the concentration of lead ions. The observed behaviour may indicate that excessive Pb(II) concentrations lead to the formation of a thicker, less homogeneous or more passive lead film on the electrode surface, which affects the tungsten adsorption conditions and reduces the detection efficiency. Based on the research carried out, it has been determined that the optimum concentration of lead ions in the solution is approximately 7.24 × 10^−5^ mol L^−1^, which ensures the maximum height of the tungsten(VI) signal while maintaining good peak quality. Exceeding this value does not provide any analytical advantage and may result in a deterioration of the electrode response.

### 3.3. Impact of Simultaneous Lead Film Formation and Tungsten Accumulation

Preliminary experiments showed that using a two-step procedure involving separate lead film deposition followed by tungsten accumulation resulted in a significantly lower analytical signal. By contrast, the simultaneous in situ formation of the lead film and tungsten accumulation produced a more intense and better-defined peak. Therefore, this study adopted the simultaneous accumulation strategy for further measurements.

The first step in optimizing the simultaneous generation of lead film and tungsten(VI) accumulation was to investigate the influence of the deposition potential on the analytical signal level. Measurements were made in the potential range −1.5 V to −0.5 V relative to the Ag/AgCl reference electrode, with a constant accumulation time of 80 s. The results obtained showed that the signal intensity increased as the potential shifted towards less negative values, reaching a maximum at −0.7 V. Further increasing the potential towards positive values resulted in a sharp decrease in signal height, which may be related to inefficient lead film deposition or limited analyte accumulation. Based on the measurements, a potential of −0.7 V was considered optimal for simultaneous deposition of the Pb(II) film and adsorption of tungsten(VI) ions, as it gave the highest current value while maintaining good analytical peak quality.

**Figure 4:**
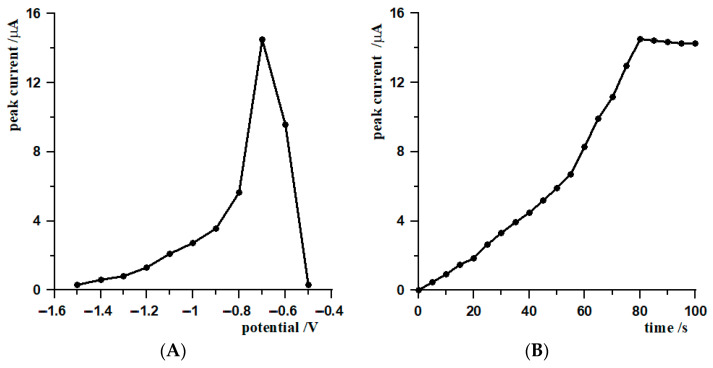
(**A**) The effect of deposition potential and (**B**) deposition time on the peak current response of 5 × 10^−7^ mol L^−1^ W(VI) at the MWCNTs/SGCE electrode. Unless otherwise stated, measurements were performed under the following conditions: accumulation time 80 s, deposition potential −0.7 V; frequency 400 Hz, amplitude 40 mV, and step potential 8 mV.

After optimizing the accumulation potential, the influence of the accumulation time on the analytical signal of tungsten(VI) was analyzed. Measurements were taken between 0 and 100 s at a constant potential of −0.7 V. The results obtained showed a systematic increase in peak current with increasing accumulation time, with a significant increase in signal observed up to approximately 80 s. After this time, the signal growth rate slowed down significantly and its value stabilized, which may indicate the saturation of the electrode surface and the achievement of a dynamic equilibrium between the accumulation and desorption of tungsten ions. On the basis of the data obtained, an accumulation time of 80 s was considered to be optimal, as it ensures high sensitivity of the determination while reducing the analysis time and avoiding excessive saturation of the electrode surface. Figure 4 shows the relationship between the potential and accumulation time and the height of the analytical signal of tungsten(VI).

### 3.4. Impact of Square Wave Voltammetry Parameters

**Figure 5:**
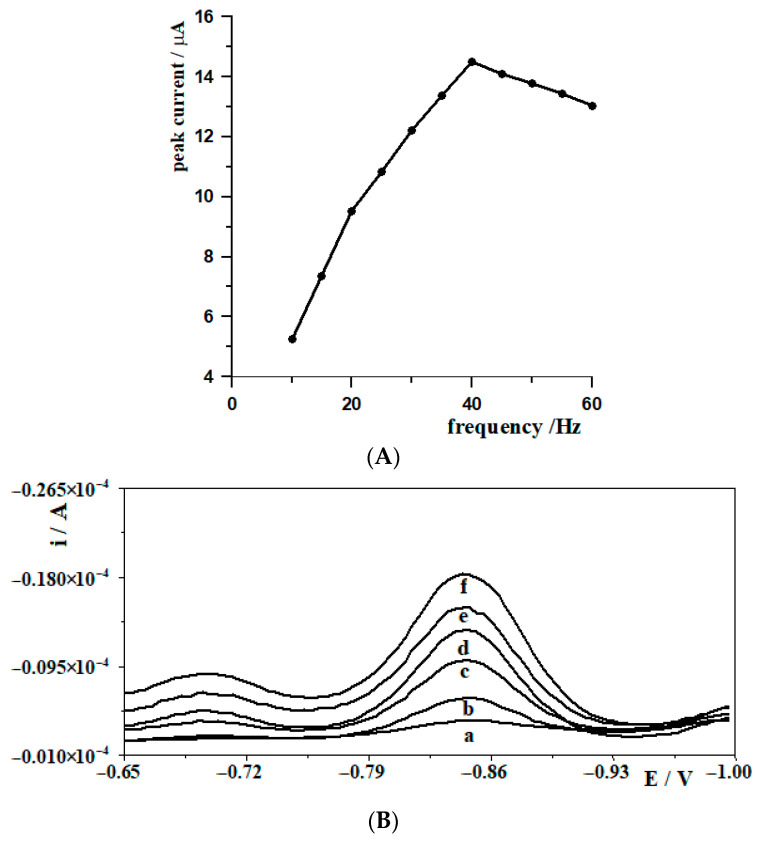
(**A**) A dependence of W(VI) peak current on the frequency. (**B**) The square wave tungsten voltammograms obtained at various frequency values: (a) 100; (b) 150; (c) 200; (d) 250; (e) 300; (f) 400. W(VI) concentration: 5 × 10^−7^ mol L^−1^.

In square wave voltammetry studies, frequency has been optimized as a key measurement parameter. The optimization was carried out in a solution containing 0.1 mol L^−1^ acetate buffer at pH 5.3, 7.24 mol L^−1^ Pb(II) and 5 × 10^−8^ mol L^−1^ W(VI). Measurements were made in the frequency range 100 Hz to 600 Hz. The signal value increases systematically from 100 Hz to 400 Hz, indicating an improvement in the electrochemical response in this range. The highest tungsten signal was observed at a frequency of 400 Hz, suggesting that this is the optimum frequency for the best measurement sensitivity. For frequencies above 400 Hz (450–600 Hz), the signal does not increase any further, but stabilizes at a similar level or even decreases slightly, which is presented in Figure 5A. This may indicate that the detection limit has been reached. In summary, the results indicate that a frequency of 400 Hz is most effective in maximizing the signal and that further increases in frequency do not provide additional benefit. Figure 5B shows the voltammograms of tungsten(VI) obtained at different pulse frequencies. These demonstrate an increase in peak current as the frequency increases. Another important parameter in these measurements is amplitude, which also affects the tungsten signal. Amplitude optimization was studied in the range 10 mV to 60 mV, at a frequency of 400 Hz and a step potential of 6 mV. The highest signal was recorded at an amplitude of 40 mV. As the amplitude pattern increased, the signal increased but the peak became very broad and therefore no higher amplitude was selected. The influence of the step potential on the peak current W(VI) was studied in the range from 2 to 12 mV, at a frequency and amplitude of 400 Hz and 40 mV respectively. Changes in the step potential in the range of 2 mV to 8 mV have a beneficial effect on the signal, increasing its intensity. A value of 8 mV is optimal as it gives the highest signal. Further increases in the step potential value will reduce the signal. A value of 8 mV is recommended as the optimum step potential parameter value for maximum analytical response. Table 1 shows the optimum parameters of the SWV technique for the electroanalytical determination of tungsten(VI).

### 3.5. Analytical Features of the Developed Method

In order to confirm the suitability of the developed analytical method for the determination of trace amounts of tungsten(VI), its validation was carried out, including the assessment of repeatability, precision, linearity and determination of the limit of detection. The tests were performed under optimized analysis conditions, i.e., at a potential of −0.7 V and an accumulation time of 80 s.

One of the key elements of the validation was the assessment of the repeatability of the analytical signal. Five peak current measurements were performed for tungsten(VI) solutions at concentrations of 5 × 10^−9^ mol L^−1^ and 1 × 10^−7^ mol L^−1^. The values obtained showed good repeatability, with relative standard deviations (RSD) of 2.97% and 2.49%, respectively. These values confirm the high precision of the method in the concentration range analyzed.

The linearity of the analytical response was evaluated based on the relationship between the concentration of tungsten(VI) and the peak current. A calibration curve was constructed in the range 7 × 10^−10^ mol L^−1^ to 7 × 10^−7^ mol L^−1^. The relationship obtained was linear, as confirmed by the high coefficient of determination (R^2^ = 0.9982). The regression equation is y = 3 × 10^7^x + 0.3969, where y is the peak height (µA) and x is the concentration of W(VI) (mol L^−1^). The limit of detection (LOD) was estimated as three times the standard deviation for the lowest studied W(VI) concentration and was equal to 3 × 10^−10^ mol L^−1^. The results obtained confirm that the developed method is characterized by high sensitivity, repeatability and a wide linearity range, which allows it to be used for the quantitative determination of tungsten(VI) in real samples.

Compared to other voltammetric techniques described in the literature, the developed method, based on the electrochemical formation of a lead film on the MWCNT/SGCE electrode, is characterized by its simplicity, high repeatability and low detection limit. For comparison, in the CSV method using a fixed bismuth microelectrode for the determination of tungsten(VI), the detection limit was 1.3 × 10^−9^ mol L^−1^ [46]. The results obtained indicate that the use of the MWCNTs/SGCE electrode made it possible to reduce the detection limit by almost one order of magnitude, which is a significant advantage of the developed analytical procedure. An important advantage of the developed method is that it does not require the use of complexing ligands, which greatly simplifies the analytical procedure and reduces the risk of additional sources of error. Methods with even lower detection limits for tungsten are also described in the literature. An example is a procedure using a HgAgFE amalgam electrode, for which the detection limit was 1.1 × 10^−12^ mol L^−1^ [51]. However, it should be noted that the use of mercury electrodes has significant practical limitations. Due to the toxicity of mercury, this type of electrode is currently being phased out of laboratory use. In addition, the method described requires the use of a complexing agent, which makes it more complex and less user-friendly. Therefore, the method we have developed is a competitive alternative that combines high sensitivity with a simplified, repeatable and safe method for the determination of tungsten(VI) in accordance with current trace analysis requirements, chemical safety standards and green chemistry.

### 3.6. Assessment of Interference Effects

Under optimized experimental conditions, the selectivity of the developed voltammetric method was evaluated by investigating the influence of selected potential interfering ions. A 100-fold excess of Cu(II), Ni(II), Co(II), Zn(II), Mg(II), Mn(II), Bi(III), As(III), Fe(III), Al(III) and Sb(III) ions was added to a solution containing tungsten(VI) at a concentration of 5 × 10^−7^ mol L^−1^. The tolerance limit was set at ±5.0%. No significant effect on the peak current of tungsten(VI) was found for any of the ions listed, indicating the high selectivity of the method with respect to these interferents.

The work also investigated in detail the influence of Sn(II), V(V), Ti(IV), Cr(VI) and Mo(VI) ions, which, as reported in the literature, had a negative effect on tungsten determinations in other voltammetric procedures. The obtained results are presented in Table 2. A 100-fold excess of V(V) reduced the tungsten signal by 75%, which is comparable to previously reported data [52]. Titanium caused a 35% decrease in signal intensity, which is lower than that reported in [52], suggesting improved tolerance in the present system. Molybdenum ions reduced the analytical signal by 58%, confirming their significant interfering effect, consistent with earlier findings [51]. Tin(II) exhibited the smallest effect among these ions, causing a 23% decrease in peak current. The results obtained confirm that the proposed method can be successfully applied to the analysis of real samples with a complex matrix, provided that the presence of specific interfering ions is controlled or determined.

The influence of selected organic substances commonly present in natural waters was investigated by adding Triton X-100, cetyltrimethylammonium bromide (CTAB), humic acids (HA), fulvic acids (FA), and natural organic matter (NOM) to solutions containing W(VI). Of the interferents tested, the greatest interference was observed with the surfactant Triton X-100, which at concentrations of 3 ppm and 5 ppm caused a decrease in analytical signal of 20% and 80%, respectively. A significant effect of Triton X-100 was also reported in [52], where its addition at a concentration of 3 ppm caused a decrease in the tungsten signal of up to 98%. A similar effect was observed for the cationic surfactant CTAB, which caused a 65% decrease in the tungsten(VI) signal at a concentration of 1 ppm. For HA and FA, the tungsten signal decreased by 60% at their concentration of 10 ppm. NOM showed a relatively smaller interference effect at a concentration of 10 ppm, with a 30% reduction in the tungsten signal. In summary, the developed method shows a limited tolerance to the presence of selected organic substances, in particular surfactants and purified humic fractions, whose presence in the sample may lead to significant interference with the analytical signal. In the case of analyses of environmental samples, it may be recommended to purify or mineralize the samples in order to limit the influence of organic compounds on the accuracy of the determination of tungsten(VI).

### 3.7. Application to Real Sample Analysis

In order to assess the suitability of the developed analytical method for the determination of tungsten(VI) in real environmental matrices, an analysis of selected samples of tap water and water from the Bystrzyca River was carried out. The samples were not specially prepared prior to measurement. To verify the accuracy of the method, a standard addition procedure was used and the results were evaluated on the basis of recovery values and repeatability of measurements. For each analysis, 5 mL of the sample was added to a 10 mL measuring vessel, meaning the sample was diluted twice. Then, a tungsten(VI) standard was added at concentrations ranging from 5 × 10^−8^ to 5 × 10^−7^ mol L^−1^. For tap water samples, the recoveries obtained ranged from 98.9% to 104.1%, confirming good consistency of determinations and indicating the absence of significant matrix interferences. The repeatability of the determinations, expressed as relative standard deviation (RSD), ranged from 0.7% to 2.4%, with the highest values recorded for the highest concentration of standard additive. The recovery values for water samples from the Bystrzyca River were also satisfactory, ranging from 97.2% to 101.1%. Slightly higher RSD values (2.2–3.6%) may be due to the greater diversity of the river sample matrix and the presence of natural organic or inorganic interferents. The results obtained clearly confirm that the developed method can be successfully applied to the determination of tungsten(VI) in real samples without the need for prior chemical preparation of the samples. The use of a standard addition procedure allows effective compensation of the matrix effect and verification of the correctness of the determinations.

In addition to real samples, certified reference materials SPS-SW2 (surface water) and SPS-WW1 (wastewater) were used to assess the suitability of the developed analytical method. Although the matrices of these materials do not contain tungsten(VI), their complex composition makes it possible to assess the effect of the presence of several potentially interfering ions on the reliability of the determination. SPS-SW2 contains 45 elements in the concentration range 0.5 ng mL^−1^ to 2000 ng mL^−1^, while SPS-WW1 contains 13 elements in the range 60–1000 ng mL^−1^. The composition of these materials includes ions that have been shown in interference studies to interfere with the tungsten signal, including vanadium(V) and molybdenum(VI). For each analysis, 1 mL of the CRMs was added to a 10 mL measuring vessel, meaning the sample was diluted tenfold. Then, a tungsten(VI) standard was added at concentrations ranging from 5 × 10^−8^ to 5 × 10^−7^ mol L^−1^. For each measurement, 1 mL of acetate buffer (0.1 mol L^−1^, pH 5.3), 1 mL of reference material (SPS-SW2 or SPS-WW1), 1 mL of a 7.24 × 10^−5^ mol L^−1^ Pb(II) solution and 110 µL of a 2 mol L^−1^ NaOH solution were added to the vessel to neutralize the nitric acid present in the CRM matrix. The total volume of the solution was made up to 10 mL with distilled water. Measurements were performed under optimized electrochemical conditions. The standard addition method was used to assess the accuracy of the determination. The recoveries obtained ranged from 96.0% to 102.6%, indicating good assay agreement and confirming that the developed method can be successfully applied to matrices of high ionic complexity. The results obtained are summarized in Table 3.

## 4. Conclusions

An electrochemical analytical sensor was developed using, for the first time, a mixture of multi-walled carbon nanotubes (MWCNTs) and spherical glassy carbon (SGC) as the working electrode material for the determination of tungsten(VI) by cathodic stripping voltammetry. The configuration developed allowed a low detection limit of 3 × 10^−10^ mol L^−1^ and a linearity range of 7 × 10^−10^ to 7 × 10^−7^ mol L^−1^. Thanks to in situ electrode modification with lead film, the time for a single analysis was only 80 s, which may be beneficial for routine determinations. The advantage of the developed method is that there is no need to complex the ligands, which simplifies the entire analytical process and increases safety in accordance with the principles of green chemistry. The applicability of the method was verified by analyzing real water samples and certified reference materials, yielding satisfactory results. Compared to the mercury electrode, the proposed solution is characterized by a more benign environmental profile, high sensitivity and simplicity of electrode preparation. All this makes the method a competitive tool in modern trace element analysis.

## Figures and Tables

**Table 1:** Optimized square-wave voltammetry (SWV) parameters for the electroanalytical determination of tungsten (VI).

Parameters	Studied Range	Optimum Value
Frequency (f) (Hz)	100–600	400
Amplitude (α) (mV)	10–60	40
Step potential (ΔEs) (mV)	2–12	8

**Table 2:** Relative W(VI) analytical signal with and without the presence of interfering ions. W(VI) concentration was 5 × 10^−7^ mol L^−1^.

Interfering Ions	Molar Excess of Interfering Ions	Relative Signal of W(VI) (I/I_0_)
Sn(II)	100	0.23
Ti(IV)	100	0.36
V(V)	100	0.75
Mo(VI)	100	0.58
Cr(VI)	100	0.77

Abbreviations: I—Tungsten peak current after the addition of an interfering ions excess; I_0_—tungsten peak current before the addition of an interfering ions excess.

**Table 3:** The results of W(VI) determination in certified reference materials and natural sample.

Sample	W(VI) Added (nmol L^−1^)	W(VI) Found (nmol L^−1^)	Recovery (%)	RSD (*n* = 3) (%)
SPS-SW2	50.0 200.0 500.0	49.1 192.0 491.0	98.2 96.0 98.2	2.17 2.35 1.95
SPS-WW1	50.0 200.0 500.0	50.6 194.9 512.8	101.2 97.5 102.6	3.4 2.1 1.8
Tap water	50.0 200.0 500.0	49.5 208.2 512.5	98.9 104.1 102.5	2.4 0.7 1.7
Bystrzyca river water	50.0 200.0 500.0	50.6 208.0 486.0	101.1 104.0 97.2	2.8 2.2 3.6

## Data Availability

The original contributions presented in this study are included in the article. Further inquiries can be directed to the corresponding author.
